# Supplementary material for: Mapping the Role of P-gp in Multidrug Resistance: Insights from Recent Structural Studies
Source: Int J Mol Sci. 2025 Apr 28;26(9):4179. doi: 10.3390/ijms26094179 (PMC12072085; doi:10.3390/ijms26094179)
Supplement: Supplementary file 1 [file ijms-26-04179-s001.zip › ijms-3588788-supplementary.pdf]

Supplementary Materials

Table S1. Studies evaluating the association between P-gp mutations and cancer treatment outcomes. The table includes details such as drug type, cancer type, specific P-gp mutations examined, outcomes of comparison, p-values, hazard ratios (HR), odds ratio (OR), and the statistical significance of the results.

Ref.	Drug type	Cancer type	Mutation	Outcome	Comparison	P-value	HR (95% CI [1])	OR (95% CI)	Conclusion
[111]	Taxane	All types	rs1045642 (C3435T)	Response	TT vs CC			1.31 (0.80-2.15)	No SA [2]
					CT vs CC			1.27 (0.80-2.02)	No SA
					CT + TT vs CC			1.05 (0.74-1.49)	No SA
					TT vs CT + CC			0.82 (0.39-1.76)	No SA
			rs1128503 (C1236T)		TT vs CC			1.14 (0.28-4.62)	No SA
					CT vs CC			1.24 (0.67-2.32)	No SA
					CT + TT vs CC			1.22 (0.51-2.94)	No SA
					TT vs CT + CC			0.91 (0.58-1.42)	No SA
[112]	Platinum/Taxane	Epithelial ovarian carcinoma	rs1128503 (C1236T)	Overall survival			0.99 (0.92-1.06)		No SA
			rs1045642 (C3435T)				0.99 (0.95-1.02)		No SA
			rs1128503 (C1236T)	Progression-free survival			1.03 (0.98-1.09)		No SA
			rs1045642 (C3435T)				1.02 (0.99-1.05)		No SA
			rs2032582 (T2677G/A)						**SA**
			rs1128503 (C1236T)						**SA**
			rs1045642 (C3435T)						**SA**
[8]	Docetaxel and epirubicin	Breast cancer	rs1045642 (C3435T)	Progression-free survival	TT vs CC	**0.03** [3]			**SA**
					CC vs CT	>0.05			No SA
				5-year survival					No SA
[7]	Paclitaxel/Carboplatin	Ovarian cancer	rs2032582 (T2677G/A)	Progression-free survival	GA/GT vs GG	**0.004**	**0.62 (0.44-0.86)**		**SA**
					TT/TA vs GG	**0.013**	**0.6 (04-0.89)**		**SA**
					GA/GT + TT/TA vs GG	**0.002**	**0.61 (0.45-0.83)**		**SA**
			rs1045642 (C3435T)		CT vs CC	0.5	1.13 (0.79-1.62)		No SA
					TT vs CC	0.3	1.22 (0.81-1.82)		No SA
			rs1128503 (C1236T)		CT vs CC	1	1 (0.71-1.39)		No SA
					TT vs CC	0.5	1.14 (0.75-1.72)		No SA
[6]	Paclitaxel/Carboplatin	Ovarian cancer	rs2229109 (G1199A)	Progression-free survival	GA + AA vs GG	0.169	1.348 (0.880-2.064)		No SA
				Overall survival	GA + AA vs GG	0.335			No SA
			rs1128503 (C1236T)	Progression-free survival	Overall	0.075			No SA
					TT vs CC	**0.006**	**0.66 (0.491-0.889)**		**SA**
					CT vs CC	0.069	0.806 (0.638-1.017)		No SA

					TT vs CT	0.124			No SA
				Overall survival	Overall	0.119			No SA
					TT vs CC	**0.007**	**0.487 (0.288-0.823)**		**SA**
					CT vs CC	0.448	0.857 (0.576-1.277)		No SA
					TT vs CT	0.052			No SA
			rs2032582 (T2677G/A)	Progression-free survival	Overall	0.181			No SA
					TT vs GG	0.087			No SA
					GT vs GG	0.605			No SA
					TT vs GT	0.112			No SA
				Overall survival	Overall	0.148			No SA
					TT vs GG	**0.006**	**0.472 (0.275-0.81)**		**SA**
					GT vs GG	0.672	0.913 (0.598-1.393)		No SA
					TT vs GT	0.061			No SA
			rs1045642 (C3435T)	Progression-free survival	Overall	**0.018**			**SA**
					TT vs CC	**0.002**	**0.623 (0.464-0.835)**		**SA**
					CT vs CC	**0.039**	**0.756 (0.580-0.986)**		**SA**
					TT vs CT	**0.039**			**SA**
				Overall survival	Overall	**0.011**			**SA**
					TT vs CC	**0.002**	**0.443 (0.264-0.746)**		**SA**
					CT vs CC	0.079	0.674 (0.434-1.047)		No SA
					TT vs CT	**0.025**	**1.348 (0.880-2.064)**		**SA**
[110]	Cytarabine and anthracyclines	Acute myeloid leukemia	rs1128503 (C1236T)	Overall survival (4 years)	CC vs CT + TT	**0.02**		**1.47 (1.07-2.01)**	**SA**
					CC + CT vs TT	0.66		1.09 (0.74-1.60)	No SA
			rs2032582 (T2677G/A)	Overall survival (3-4 years)	GG vs GT + TT	**0.04**		**1.37 (1.01-1.86)**	**SA**
					GG + GT vs TT	0.55		1.11 (0.78-1.60)	No SA
			rs1045642 (C3435T)	Overall survival (3 years)	CC vs CT + TT	**0.03**		**1.41 (1.03-1.94)**	**SA**
					CC + CT vs TT	0.16		1.25 (0.92-1.71)	No SA
				Overall survival (4-5 years)	CC vs CT + TT	**0.02**		**1.42 (1.05-1.91)**	**SA**
					CC + CT vs TT	**0.018**		**1.22 (0.91-1.65)**	**SA**
[118]	Chemotherapeutics	Esophageal cancer	rs1045642 (C3435T)	Overall survival			**0.57 (0.37-0.87)**		**SA**
							**0.51 (0.32-0.81)**		**SA**
[119]	Chemotherapeutics	Leukemia	rs1045642 (C3435T)	Drug resistance/sensitive		0.6			No SA
			rs2032582 (T2677G/A)			1			No SA
			rs3213619 (T-129C)			1			No SA
[120]	Chemotherapeutics	Invasive epithelial ovarian cancer	rs6946119	Time to recurrence		0.83	1.02 (0.84 - 1.25)		No SA
			rs1055302			0.2	0.84 (0.64 - 1.10)		No SA

			rs17064			0.28	0.82 (0.57 - 1.17)		No SA
			rs2235048			0.54	1.05 (0.89 - 1.24)		No SA
			rs6949448			0.52	0.95 (0.80 - 1.12)		No SA
			rs7787082			0.14	0.84 (0.66 - 1.06)		No SA
			rs11760837			**0.03**	**0.72 (0.53 - 0.97)**		**SA**
			rs10274587			**0.03**	**0.72 (0.53 - 0.97)**		**SA**
			rs12720066			0.87	0.97 (0.65 - 1.43)		No SA
			rs1922242			0.06	1.17 (0.99 - 1.37)		No SA
			rs2091766			0.24	1.10 (0.94 - 1.29)		No SA
			rs2235033			0.09	0.86 (0.73 - 1.02)		No SA
			rs2032588			**0.03**	**0.67 (0.47 - 0.97)**		**SA**
			rs1128503 (C1236T)			0.95	1.01 (0.85 - 1.19)		No SA
			rs2235023			**0.003**	**0.58 (0.40 - 0.82)**		**SA**
			rs13237132			**0.02**	**1.21 (1.03 - 1.44)**		**SA**
			rs12334183			**0.0005**	**0.65 (0.51 - 0.83)**		**SA**
			rs10264990			**0.01**	**1.24 (1.05 - 1.47)**		**SA**
			rs1989830			**0.03**	**0.82 (0.68 - 0.98)**		**SA**
			rs1202172			**0.03**	**0.82 (0.68 - 0.98)**		**SA**
			rs17327442			0.33	1.12 (0.89 - 1.40)		No SA
			rs1202184			0.19	1.12 (0.95 - 1.32)		No SA
			rs1211152			0.68	0.94 (0.71 - 1.25)		No SA
			rs17327624			0.37	1.10 (0.89 - 1.34)		No SA
			rs13229143			**0.03**	**0.83 (0.70 - 0.99)**		**SA**
			rs12535512			0.75	1.03 (0.87 - 1.22)		No SA
			rs3789243			0.23	1.11 (0.94 - 1.31)		No SA
			rs2214102			0.27	0.85 (0.64 - 1.13)		No SA
			rs4728709			0.18	1.22 (0.92 - 1.62)		No SA
			rs4148732			**0.04**	**1.28 (1.01 - 1.60)**		**SA**

			rs13233308			0.44	0.94 (0.79 - 1.10)		No SA
			rs2157926			0.18	1.22 (0.92 - 1.62)		No SA
			rs10246878			0.89	0.99 (0.81 - 1.21)		No SA
[121]	Chemo therape utics	Epithelial ovarian carcinoma	rs7793196	Progression-free survival		0.47	1.09 (0.86–1.37)		No SA
			rs6946119			0.175	1.15 (0.94–1.42)		No SA
			rs6979885			**0.033**	**1.26 (1.02–1.56)**		**SA**
			rs2235048			**0.015**	**1.26 (1.05–1.52)**		**SA**
			rs1045642 (C3435T)			**0.022**	**1.25 (1.03–1.51)**		**SA**
			rs2032582 (T2677G/A)			**0.001**	**0.74 (0.62–0.89)**		**SA**
			rs4148738			**0.015**	**0.79 (0.65–0.96)**		**SA**
			rs10276603			0.642	1.07 (0.81–1.41)		No SA
			rs2091766			0.272	1.12 (0.91–1.38)		No SA
			rs1128503 (C1236T)			0.119	0.85 (0.70–1.04)		No SA
			rs12704364			0.13	1.17 (0.96–1.42)		No SA
			rs956825			0.686	1.04 (0.85–1.28)		No SA
			rs10260862			0.23	1.15 (0.91–1.46)		No SA
			rs10264990			**0.01**	**1.33 (1.07–1.65)**		**SA**
			rs1202174			0.096	1.19 (0.97–1.45)		No SA
			rs17327442			0.345	1.15 (0.86–1.55)		No SA
			rs1202184			**0.03**	**1.24 (1.02–1.51)**		**SA**
			rs1211152			0.21	1.3 (0.86–1.96)		No SA
			rs17327624			0.871	0.98 (0.76–1.26)		No SA
			rs2188526			**0.009**	**0.77 (0.63–0.94)**		**SA**
			rs13233308			**0.01**	**0.78 (0.64–0.94)**		**SA**
[122]	Dasatin ib	Chronic myeloid leukemia	rs7787082	Cytogenic response				0.20 (0.06-0.66)	No SA
			rs3842	Overall survival		**0.012**	**1.84 (1.01-3.33)**		**SA**
			rs2235023			**0.027**	**2.28 (1.03-5.02)**		**SA**
			rs2235023	Progression-free survival		0.11	2.49 (1.13-5.50)		No SA
			rs22114102			**0.028**	**1.90 (1.00-3.63)**		**SA**
[123]	Cabazit axel	Metastatic castration-resistant prostate cancer	rs1202179	Progression-free survival	No statistical data				No SA
			rs1202172						No SA
			rs1202171						No SA
			rs4148733						No SA
			rs1202186						No SA
			rs1202184						No SA
			rs17327624						No SA
			rs3789243						No SA
			rs9282564 (A61G)						No SA
			rs2214102						No SA
			rs6949448						No SA
			rs2235067						No SA
			rs2235040						No SA
			rs2235046						No SA
			rs2235013						No SA
			rs2235035						No SA
			rs2235033						No SA

			rs1128503 (C1236T)						No SA
			rs10276036						No SA
			rs1922240						No SA
			rs1202170						No SA
			rs1045642 (C3435T)						No SA
			rs1202179	Overall survival	No statistical data				No SA
			rs1202172						No SA
			rs1202171						No SA
			rs4148733						No SA
			rs1202186						No SA
			rs1202184						No SA
			rs17327624						No SA
			rs3789243						No SA
			rs9282564 (A61G)						No SA
			rs2214102						No SA
			rs6949448						No SA
			rs2235067						No SA
			rs2235040						No SA
			rs2235046						No SA
			rs2235013						No SA
			rs2235035						No SA
			rs2235033						No SA
			rs1128503 (C1236T)						No SA
			rs10276036						No SA
			rs1922240						No SA
			rs1202170						No SA
			rs1045642 (C3435T)						No SA
[124]	Anthracyclines/mitoxantrone + Cytarabine	Acute myeloid leukemia	rs2229109 (G1199A)	Overall survival		0.372	1.48 (0.63–3.53)		No SA
						0.542	1.31 (0.55–3.08)		No SA
			rs1128503 (C1236T)		CT vs CC	**0.007**	**0.32 (0.14–0.73)**		**SA**
					TT vs CC	**0.005**	**0.24 (0.09–0.65)**		**SA**
			rs1045642 (C3435T)		CT vs CC	0.806	1.12 (0.46–2.69)		No SA
						0.34	1.55 (0.63–3.84)		No SA
					TT vs CC	0.428	1.55 (0.53–4.57)		No SA
						0.425	1.55 (0.53–4.55)		No SA
			rs2032582 (T2677G/A)		GT vs GG	**0.001**	**0.25 (0.11–0.58)**		**SA**
					TT vs GG	**0.003**	**0.22 (0.08–0.60)**		**SA**
			A1308T						Excluded due to minimum allele frequency
[125]	Idarubicin + cytarabine + etoposide	Acute myeloid leukemia	C193T	Overall survival	TC vs CC	0.02	RR: 2.1 (1.1-4.1)		No SA
			I44M						No SA
[126]	Gemtuzumab		rs3842						Excluded due to

	ozoga micin	Acute myeloid leukemia							minimum allele frequency
			rs9282564 (A61G)						Excluded due to minimum allele frequency
			rs2229107 (T3421A)						Excluded due to minimum allele frequency
	Chemo therape utics		rs1045642 (C3435T)	Overall survival	CC vs CT vs TT	0.323			No SA
					CC vs CT + TT	0.371			No SA
				Event-free survival	CC vs CT vs TT	0.633			No SA
					CC vs CT + TT	0.466			No SA
				Disease-free survival	CC vs CT vs TT	0.441			No SA
					CC vs CT + TT	0.472			No SA
	Chemo therape utics + Gemtu zumab ozoga micin			Overall survival	CC vs CT vs TT	0.123			No SA
					CC vs CT + TT	0.068			No SA
				Event-free survival	CC vs CT vs TT	0.073			No SA
					CC vs CT + TT	**0.022**			**SA**
				Disease-free survival	CC vs CT vs TT	**0.024**			**SA**
					CC vs CT + TT	**0.007**			**SA**
	Chemo therape utics		rs2235015	Overall survival	GG vs GT vs TT	0.848			No SA
					GG + GT vs TT	0.954			No SA
				Event-free survival	GG vs GT vs TT	0.892			No SA
					GG + GT vs TT	0.644			No SA
				Disease-free survival	GG vs GT vs TT	0.934			No SA
					GG + GT vs TT	0.719			No SA
	Chemo therape utics + Gemtu zumab ozoga micin		rs2235015	Overall survival	GG vs GT vs TT	0.45			No SA
					GG + GT vs TT	0.247			No SA
				Event-free survival	GG vs GT vs TT	0.303			No SA
					GG + GT vs TT	0.156			No SA
				Disease-free survival	GG vs GT vs TT	0.286			No SA
					GG + GT vs TT	0.118			No SA
	Chemo therape utics		rs2235033	No statistical data					No SA
			rs1128503 (C1236T)						No SA
			rs1922242						No SA

			rs1922240						No SA
			rs1989830						No SA
			rs2032582 (T2677G/A)						No SA
			rs2235040						No SA
[127]	Chemo therape utics	Multiple myeloma	rs7789645						No SA
			rs998671						No SA
			rs3842						No SA
			rs17064						No SA
			rs6979885						No SA
			rs1045642 (C3435T)						No SA
			rs7787082						No SA
			rs12720066						No SA
			rs1922242						No SA
			rs868755						No SA
			rs2235023						No SA
			rs10256836						No SA
			rs12334183	No statistical data					No SA
			rs10264990						No SA
			rs1202172						No SA
			rs17327442						No SA
			rs1202184						No SA
			rs17327624						No SA
			rs3789243						No SA
			rs2235074						No SA
			rs9282564 (A61G)						No SA
			rs2214102						No SA
			rs13233308						No SA
			rs10276499						No SA
			rs10267099						No SA
			rs2235013	Overall survival	Codominant model (heterozygous)	0.421	1.21 (0.75-1.96)		No SA
					Codominant model (homozygous)	0.072	0·59 (0·33–1·08)		No SA
					Dominant model	0.767	0·93 (0·59–1·47)		No SA
					Recessive model	**0.006**	**0·52 (0·33–0·83)**		**SA**
				Progression-free survival	Codominant model (heterozygous)	0.471	1·26 (0·66–2·42)		No SA
					Codominant model (homozygous)	0.676	1·15 (0·59–2·25)		No SA
					Dominant model	0.508	1·21 (0·68–2·15)		No SA
					Recessive model	0.949	1·01 (0·58–1·79)		No SA

[1] CI: confidence interval.

[2] SA: Significant association.

[3] Bold numbers indicate p value of less than 0.05.

Table S2. Summary of common interacting residues of P-gp with elacridar, tariquidar and zosuquidar.

Interacting residues of P-gp	Zosuquidar	Elacridar	Tariquidar
L65	✓	✓	✓
M69	✗	✓	✓
W232	✓	✓	✓
L236	✓	✓	✗
I299	✓	✓	✓
F303	✓	✓	✓
I306	✓	✓	✓
Y310	✓	✓	✓
F336	✓	✓	✓
I340	✓	✓	✓
F343	✓	✓	✓
Q347	✓	✗	✓
Q725	✓	✓	✓
F728	✗	✓	✓
F770	✓	✓	✗
L879	✓	✗	✓
M949	✓	✓	✓
Y953	✓	✓	✓
F983	✓	✓	✓
M986	✓	✓	✓
Q990	✓	✗	✓
F994	✓	✓	✗
